# Non-thyroidal Illness Syndrome and Clinical Outcomes in Critically Ill ICU Patients: A Prospective Cohort Study

**DOI:** 10.7759/cureus.110424

**Published:** 2026-06-07

**Authors:** Razan Rabi, Imad Ismail, Mays Safa, Anas Odeh, Khair Marmash, Dina Abugaber

**Affiliations:** 1 Internal Medicine, An-Najah National University Hospital, Nablus, PSE; 2 Medicine, An-Najah National University Hospital, Nablus, PSE; 3 Department of Medicine, Cleveland Clinic, Cleveland, USA; 4 Faculty of Medicine and Health Sciences, An-Najah National University, Nablus, PSE

**Keywords:** critical illness, intensive care unit, low ft3, mortality, non-thyroidal illness syndrome, thyroid hormones

## Abstract

Background

Non-thyroidal illness syndrome (NTIS) is a common condition in critically ill patients, yet its prognostic value remains unclear. This study aimed to assess the incidence of NTIS among critically ill patients and evaluate its association with clinical outcomes and mortality.

Methods

A prospective cohort study was conducted in a tertiary hospital between 2019 and 2025. Adult intensive care unit (ICU) patients without known thyroid disease were included. Thyroid function tests, including thyroid-stimulating hormone (TSH), free thyroxine (FT4), and free triiodothyronine (FT3), were measured within 24 hours of admission. Clinical data, comorbidities, and outcomes were recorded. Patients were classified as having low FT3 (<3.0 nmol/L) or normal FT3 (≥3.0 nmol/L). Logistic regression and receiver operating characteristic (ROC) analysis were used to evaluate the prognostic performance of thyroid hormones, with survival used as the reference outcome.

Results

Among 180 patients (mean age = 57.7 ± 18 years; 111/180 (62%) male), 93/180 (52%) had NTIS. Patients with low FT3 were older and had higher rates of diabetes, cardiovascular, and pulmonary diseases (p < 0.05). They more frequently developed shock, respiratory failure, and required mechanical ventilation. ICU mortality was significantly higher among the low FT3 group (37/93 (43%) vs. 7/85 (7.5%), p < 0.001). After adjustment for age, comorbidities, and illness severity, both low FT3 (OR = 0.23, 95% CI = 0.07-0.79; p = 0.007) and low FT4 (OR = 0.80, 95% CI = 0.65-0.99; p = 0.03) remained independently associated with mortality, whereas TSH showed no significant association. ROC analysis demonstrated that FT3 had the strongest association with survival (area under the curve = 0.208, p < 0.001), reflecting an inverse relationship when survival was used as the reference outcome.

Conclusion

NTIS is highly prevalent in critically ill patients and is associated with increased mortality and adverse outcomes. Low FT3, in particular, is associated with disease severity and may serve as a prognostic marker in the ICU; however, these findings reflect association rather than causation.

## Introduction

Recent studies have found that alterations in thyroid hormones are closely linked to mortality and overall prognosis among critically ill patients. These changes in thyroid hormones are referred to as euthyroid sick syndrome, non-thyroidal illness syndrome (NTIS), or low free triiodothyronine (FT3) syndrome [1,2]. This results from disruptions in the hypothalamic-pituitary-thyroid axis, characterized by decreased FT3 levels, variable free thyroxine (FT4) changes, and typically normal or decreased thyroid-stimulating hormone (TSH) levels. The changes in serum FT3 and reverse FT3 (rT3) levels depend on the severity of the illness. In mild to moderate NTIS, FT4 and TSH levels are usually normal with low free FT3 and increased rT3, but in more severe or long-lasting illnesses, they are often low [1,2].

One of the major mechanisms behind these thyroidal hormonal alterations is the altered activity of deiodinases, the enzymes that regulate the metabolism of thyroid hormones. Decreased activity of type 1 and type 2 deiodinases in peripheral tissues reduces the conversion of FT4 to the bioactive form FT3, while the increased activity of deiodinase type 3 leads to the degradation of FT3 and FT4 to inactive metabolites such as rT3 [2,3]. The low FT3 levels are often caused by proinflammatory cytokines (such as IL-6 and tumor necrosis factor-alpha) and oxidative stress, especially in conditions such as sepsis, renal failure, or multi-organ failure, which contribute to the low FT3 state seen in NTIS [1,3,4].

Observational studies have shown a significant association between these thyroid profile abnormalities and increased mortality risk among intensive care unit (ICU) patients, lending support for the use of thyroid hormones as prognostic markers in critical care settings [2,5]. The prognostic value of thyroid profiles (i.e., low FT3 and abnormal TSH) has also been associated with disease severity and outcomes among critically ill populations [6,7]. In addition, other studies have shown that non-thyroidal illness is common in more inclusive ICUs and have identified risk factors such as sepsis and mechanical ventilation, which may aggravate thyroid dysfunction [1,3]. Understanding how thyroid function relates to mortality in the ICU is important to establish its clinical use as a prognostic indicator and a possible therapeutic target. Therefore, the present study aims to assess the incidence of NTIS among ICU patients and to examine its association with clinical outcomes, including disease severity and mortality. Furthermore, the study seeks to identify potential risk factors that contribute to the development of NTIS in critically ill patients and to clarify its prognostic significance and potential role as a therapeutic target in critical care settings.

This article was previously posted to the medRxiv preprint server in October, 2025.

## Materials and methods

Study design

This study was a prospective cohort study of critically ill patients who were admitted to either the medical or surgical ICU sections at An-Najah National University Hospital. The calculated minimum sample size was 176, based on an expected prevalence of 30% thyroid dysfunction, 80% power, and a 95% confidence interval. Inclusion criteria were critically ill patients aged 18-90 years or older admitted to the ICU. Exclusion criteria included patients with a known thyroid disease, patients being treated for hypo- or hyperthyroidism in the past, and pregnant women. The study period (2019-2025) reflects the extended recruitment timeframe required to achieve the calculated sample size. Patient enrollment was continuous but dependent on ICU admissions and the availability of laboratory testing during this period.

Data collection

Thyroid function tests (TSH, FT3, FT4) were evaluated in the first 24 hours of ICU admission. In addition to thyroid function tests, comprehensive data were collected on various patient-related variables. Demographics such as age, gender, and body mass index (BMI) were collected at the time of ICU admission. Data on comorbidities, including diabetes, hypertension, cardiovascular disease, and malignancies, as well as the reasons for hospital and ICU admission and the presence of shock, respiratory failure, or a requirement for mechanical ventilation, were also collected. Blood tests obtained at ICU admission included white blood cell count (WBC), hemoglobin (HGB), platelet count (PLT), C-reactive protein (CRP), creatinine, potassium (K), sodium (Na), and international normalized ratio (INR). Outcomes were recorded based on mortality or discharge status during the ICU stay. Moreover, the duration of mechanical ventilation was observed along with the occurrence of acute kidney injury (AKI), shock, or respiratory failure post-ICU admission. Reference laboratory ranges were as follows: TSH = 0.4-4.0 mIU/L; FT3 = 3.0-6.5 nmol/L; and FT4 = 12-22 nmol/L.

The study was approved by the Institutional Review Board of An-Najah National University Hospital (Reference No.: Med.June.2024/10). Written informed consent was obtained from all participants or their legal surrogates. This study was conducted in accordance with the Strengthening the Reporting of Observational Studies in Epidemiology (STROBE) guidelines [8]. No external funding was received for this study, and the authors declare no conflicts of interest.

Definitions

Thyroid dysfunction was defined as any abnormality in thyroid hormone levels (TSH, FT3, and FT4) relative to the reference ranges for critically ill patients. Shock was recognized as a state of inadequate blood flow that causes inadequate tissue oxygenation, which requires vasopressor support according to the Surviving Sepsis Campaign definition [9]. Respiratory failure was defined as an inappropriate gas exchange characterized by either or both hypoxemia and hypercapnia, and AKI was defined as an unexpected increase in renal dysfunction characterized by an increase in serum creatinine and/or decrease in urine output based on the definition of Kidney Disease Improving Global Outcomes (KDIGO) [10].

NTIS was diagnosed based on an FT3 level below the lower limit of the laboratory reference range (<3.0 nmol/L) in the absence of a known thyroid disorder, together with normal or low TSH and FT4 levels.

Statistical analysis

Statistical tests were used for variables to analyze the data accordingly to assess the association between variables; for numerical variables, the independent T-test was used. Chi-square and Fisher's exact tests were conducted with categorical variables. Almost all comparisons between the study groups were assessed, adjusting for confounders with logistic regression. Multivariable logistic regression was performed using ICU mortality as the dependent variable. Covariates were selected based on clinical relevance and significant associations identified during univariate analysis. The confounders included variables such as the presence of comorbidities (e.g., diabetes and hypertension), illness severity, and ICU treatments.

For analytical purposes, patients were classified into two groups based on their serum FT3 levels, i.e., low FT3 (<3.0 nmol/L) and normal FT3 (≥3.0 nmol/L), using the lower limit of the laboratory reference range as the clinical cut-off. A receiver operating characteristic (ROC) curve analysis was then performed to assess the diagnostic performance of various thyroid hormones, including FT3, FT4, and TSH, in identifying NTIS. The ROC analysis provided the optimal cut-off values for each hormone, including an FT3 threshold of 1.6 nmol/L. Area under the curve (AUC) values were calculated for FT3, FT4, and TSH to evaluate their sensitivity, specificity, and overall diagnostic accuracy. While this lower threshold demonstrated improved specificity, the standard reference value was retained for group comparisons to maintain clinical relevance and interpretability. The multivariable model was adjusted for age, diabetes mellitus, cardiovascular disease, sepsis, shock, respiratory failure, and mechanical ventilation. Odds ratios (ORs) with 95% confidence intervals (CIs) were reported. All analyses were performed using SPSS Statistics version 25 (IBM Corp., Armonk, NY).

## Results

Of the total sample of 180 ICU patients, the mean age was 57.7 ± 18 years, with 111 (62%) males. Comorbidities included diabetes (68, 38%), hypertension (87, 49%), cardiovascular disease (66, 36%), chronic pulmonary disease (25, 14%), malignancy (63, 35%), chronic kidney disease (33, 18%), end-stage renal disease (13, 7%), chronic liver disease (10, 5.6%), and smoking (50, 28%). Patients were admitted to the surgical ICU (108, 60%) or the medical ICU (72, 40%), with 66 (36%) having sepsis, 64 (36%) being in shock, 107 (59%) with respiratory failure, and 54 (30%) requiring mechanical ventilation. Baseline demographic and clinical characteristics are summarized in Table 1.

**Table 1:** Baseline characteristics and comparisons of critically ill ICU patients by thyroid function status. FT3, free triiodothyronine; SD, standard deviation; ICU, intensive care unit; WBC, white blood cell count; HGB, hemoglobin; PLT, platelet count; CRP, C-reactive protein; INR, international normalized ratio; χ², chi-square; df, degrees of freedom; Cramer’s V, effect size.

Variables	Total (n = 180)	Low FT3 (n = 93)	Normal FT3 (n = 87)	P-value	χ² (df)	Cramer’s V
Age, mean ± SD	57.7 ± 18	61.8 ± 18	54 ± 18	0.004	-	-
Gender, male, n (%)	111 (62)	49 (57)	60 (64)	0.4	0.98 (1)	0.07
Comorbidities						
Diabetes	68 (38)	39 (46)	29 (31)	0.04	2.32 (1)	0.11
Hypertension	87 (49)	46 (54)	40 (43)	0.16	5.74 (1)	0.18
Cardiovascular disease	66 (36)	39 (45)	26 (28)	0.013	11.6 (1)	0.25
Chronic pulmonary disease	25 (14)	19 (22)	5 (5.4)	0.001	2.21 (1)	0.11
Malignancy	63 (35)	36 (42)	26 (28)	0.06	2.32 (1)	0.11
Hematological	17 (27)	11 (30)	6 (23)	0.5	0.45 (1)	0.12
Chronic kidney disease	33 (18)	20 (23)	13 (14)	0.1	2.41 (1)	0.12
End-stage renal disease	13 (7)	6 (7.1)	7 (7.5)	0.8	0.02 (1)	0.01
Chronic liver disease	10 (5.6)	7 (8)	3 (3)	0.15	1.74 (1)	0.10
Smoker	50 (28)	27 ( 32)	23 (25)	0.3	1.19 (1)	0.08
Location, Surgical ICU	108 (60)	38 (54)	67 (72)	<0.001	9.21 (1)	0.29
Medical ICU	72 (40)	45 (52)	26 (28)		0.23	0.23
Baseline status						
Sepsis	66 (36)	47 (55.3)	19 (20)	<0.001	24.58 (1)	0.37
Shock	64 (36)	48 (56)	16 (17)	<0.001	29.21 (1)	0.40
Septic	31 (47)	25 (52)	6 (33)	<0.001	11.62 (1)	0.25
Hemorrhagic	15 (23)	11 (23)	4 (22)	0.057	3.58 (1)	0.14
Cardiogenic	11 (16)	6 (12)	5 (27)	0.6	0.27 (1)	0.04
Mixed	5 (7)	5 (10)	-		10.44 (1)	
Respiratory failure	107 (59)	62 (72)	44 (47)	0.001	7.23 (1)	0.24
Mechanical ventilated	54 (30)	34 (40)	20 (21.5)	0.007	24.58 (1)	0.20
Baseline labs						
WBC (×10⁹/L)	13 ± 8.3	13.1 ± 9.3	12.9 ± 7.5	0.9	-	-
HGB (g/dL)	11 ± 2.6	10 ± 2.2	11.9 ± 2.5	<0.001	-	-
PLT (×10⁹/L)	228 ± 128	223 ± 147	232 ± 110	0.6	-	-
Creatinine (mg/dL)	1.5 ± 1.7	1.78 ± 1.9	1.3 ± 1.5	0.06	-	-
CRP (mg/L)	82 ± 110	116.7 ± 116	48 ± 94	<0.01	-	-
INR	1.3 ± 0.4	1.4 ± 0.5	1.3 ± 0.5	0.01	-	-

Respiratory failure and postoperative conditions were the most common reasons for ICU admission, accounting for 39 (22%) and 40 (22%) patients, respectively. Septic shock was present in 25 (14%) patients, while hemorrhagic shock and brain hemorrhage accounted for 30 (16.7%) and 13 (7%) cases, respectively. Gastrointestinal bleeding, cardiogenic shock, and stroke were less frequent, occurring in five (3%), three (1.7%), and seven (4%) patients, respectively. Other unspecified causes accounted for 33 (17%) of the cases.

Univariate and multivariate analyses revealed a strong association between thyroid hormone levels and mortality. Mean FT3 and FT4 concentrations were significantly lower among non-survivors compared to survivors (FT3: 2.3 ± 0.8 vs. 3.6 ± 1.9, p-value < 0.01; FT4: 16 ± 6.1 vs. 19 ± 5.2, p-value = 0.01). After adjustment for age, comorbidities, and critical illness factors, both FT3 (adjusted p = 0.007, OR = 0.23, 95% CI = 0.07-0.79) and FT4 (adjusted p = 0.03, OR = 0.98, 95% CI = 0.8-0.99) remained independently associated with mortality, indicating that lower hormone levels predicted poorer outcomes. In contrast, TSH levels did not differ significantly between survivors and non-survivors (p-value = 0.7), suggesting that pituitary feedback remains blunted during severe illness.

The ROC curve analysis further demonstrated that low FT3 had the strongest inverse correlation with survival (AUC = 0.208, p < 0.001), followed by FT4 (AUC = 0.361, p-value = 0.006), whereas TSH failed to predict mortality (AUC = 0.522, p-value = 0.664). These data confirm that thyroidal suppression, particularly reduced FT3, reflects metabolic and inflammatory stress intensity and can serve as a biochemical marker of poor prognosis in the ICU. FT3 demonstrated good discriminatory ability for mortality prediction (AUC = 0.792, calculated as 1 − AUC due to survival being used as the reference outcome). FT4 showed modest discrimination (AUC = 0.639), while TSH had no predictive value. The association between thyroid hormone levels and mortality is presented in Table 2.

**Table 2:** Univariate and multivariate analysis of thyroid hormone levels and their association with mortality. TSH, thyroid-stimulating hormone; FT3, free triiodothyronine; FT4, free thyroxine; OR, odds ratio; CI, confidence interval.

Variables	Total (n = 180)	Alive (n = 136)	Deceased (n = 44)	P-value	Adjusted p-value	OR (95% CI)
TSH (mIU/L)	3.1 ± 6.6	3.3 ± 7.4	2.8 ± 2.8	0.7	0.9	0.95 (0.92-1.01)
FT3 (nmol/L)	3.3 ± 1.8	3.6 ± 1.9	2.3 ± 0.8	<0.01	0.007	0.4 (0.23-0.79)
FT4 (nmol/L)	18 ± 5.6	19 ± 5.2	16 ± 6.1	0.01	0.03	0.89 (0.8-0.99)

Patients with low FT3 had significantly worse clinical outcomes. The overall ICU mortality rate was 44 (24%), but was markedly higher among those with low FT3 compared to patients with normal FT3 levels (37 (43%) vs. 7 (7.5%), p-value < 0.001). Similarly, 28-day mortality was substantially elevated in the low FT3 group (17 (20%) vs. 3 (3%), p-value < 0.001). The incidence of AKI was also greater among patients with low FT3 (19 (22%) vs. 10 (10.8%), p-value = 0.036), and shock occurred more frequently (12 (14%) vs. 2 (2%), p-value = 0.03), emphasizing the link between hormonal suppression and organ dysfunction.

Although differences in mechanical ventilation duration and ICU length of stay were not statistically significant, low FT3 patients tended to require longer respiratory and hemodynamic support, consistent with more severe disease. Collectively, these outcomes reinforce that NTIS is not merely a biochemical finding but a reflection of critical systemic compromise associated with higher morbidity and mortality. Clinical outcomes according to thyroid function status are summarized in Table 3.

**Table 3:** Outcomes in critically Ill patients by thyroid function status. ICU, intensive care unit; FT3, free triiodothyronine; χ², chi-square; df, degrees of freedom; Cramer’s V, effect size.

Outcome	Total (n = 180)	Low FT3 (n = 93)	Normal FT3 (n = 87)	P-value	χ² (df)	Cramer’s V
Mortality, n (%)	44 (24)	37 (43)	7 (7.5)	<0.001	22.09 (1)	0.352
28-day mortality, n (%)	20 (11)	17 (20)	3 (3)	<0.001	8.27 (1)	0.215
Mechanical ventilation, n (%)	20 (11)	12 (14)	8 (8.7)	0.3	0.25 (1)	0.037
Duration of ventilation, days	15 ± 20	11.7 ± 18	20.8 ± 48	0.4	-	-
Acute kidney injury, n (%)	29 (16)	19 (22)	10 (10.8)	0.036	1.85 (1)	0.102
Shock, n (%)	14 (8)	12 (14)	2 (2)	0.03	5.44 (1)	0.175
Respiratory failure, n (%)	30 (17)	18 (21)	12 (12.9)	0.14	0.54 (1)	0.055
ICU days	8.3 ± 17	9.1 ± 14	7.7 ± 20	0.59	-	-

**Figure 1:**
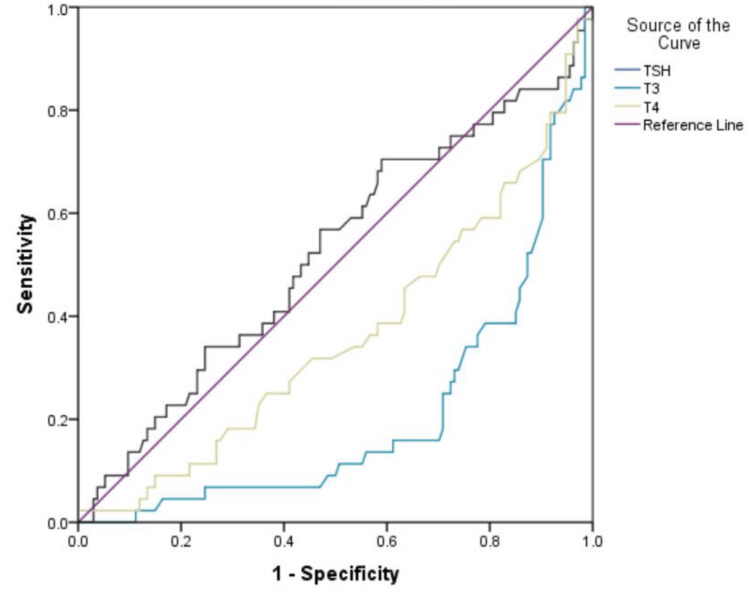
Receiver operating characteristic (ROC) curves of TSH, FT3, and FT4 for predicting mortality. TSH, thyroid-stimulating hormone; FT3, free triiodothyronine; FT4, free thyroxine.

The AUC for FT3 was 0.208 (p < 0.001), indicating that lower FT3 concentrations were strongly linked to poor survival outcomes. FT4 also showed a modest but significant predictive value (AUC = 0.361, p = 0.006), whereas TSH failed to demonstrate discriminatory performance (AUC = 0.522, p = 0.664). Because survival rather than mortality was used as the reference outcome in the ROC analysis, AUC values below 0.5 indicate an inverse relationship, whereby lower FT3 and FT4 concentrations were associated with a higher risk of death. If mortality had been used as the reference outcome, the corresponding AUC values would be greater than 0.5 while reflecting the same discriminatory performance. These findings support that depressed FT3 and, to a lesser extent, FT4 levels are reflective of disease severity and metabolic suppression during critical illness. However, the overall predictive accuracy remained limited, suggesting that thyroid hormone changes should be interpreted alongside clinical and biochemical indicators of organ dysfunction rather than as stand-alone prognostic tools. ROC curves for thyroid hormones in predicting mortality are shown in Figure 1.

## Discussion

This study demonstrated that NTIS is common among critically ill patients and that low FT3 is significantly associated with increased mortality and complications. The primary biochemical abnormalities were a low plasma triiodothyronine level, increased reverse T3, and either low or normal thyroxine and TSH levels [5,7,11-13]. NTIS is highly prevalent. A systematic review and meta-analysis that included 6,869 patients from 25 studies showed a median prevalence of 58% [14]. Other observational studies showed variable prevalence, from 17% to 54% (based on low FT3) [3,5-7,15,16], and up to 80% (general definition) [6,14,15]. The occurrence and severity of NTIS were associated with poor prognosis and higher all-cause mortality. It should be noted that the absence of validated severity indices such as Sequential Organ Failure Assessment (SOFA) or Acute Physiology and Chronic Health Evaluation II (APACHE II) limits our ability to determine whether FT3 is independently associated with mortality or primarily reflects underlying illness severity.

In this prospective cohort of 180 critically ill patients admitted to medical or surgical ICUs, the prevalence was similar to what is reported in the literature, but in the lower range (51.7%). The effect of low FT3 on outcomes was assessed. The results showed that patients with low FT3 had a significantly higher overall mortality compared to those without thyroidal illness (43% vs. 7.5%, p-value < 0.001), and the 28-day mortality was also higher (20% vs. 3%, p-value < 0.001). Both FT3 and FT4 levels were significantly associated with mortality in multivariate analysis, while TSH did not show a significant effect. Although the AUC for FT3 was below 0.5, indicating an inverse association with survival, it still reflects a strong predictive relationship between low FT3 and mortality (AUC = 0.208, p-value < 0.001). In addition, noting that patients with low FT3 were significantly older and more likely to develop acute kidney injury and shock.

These findings are similar to the literature in which low FT3 was associated with worse outcomes in critical illness. Since AUC values below 0.5 indicate an inverse relationship, this suggests that lower FT3 levels strongly correlate with mortality rather than protective outcomes. In a systematic review, it was found that FT3 and FT4 levels were lower in non-survivors than in survivors, and NTIS was independently associated with increased risk of mortality (OR = 2.21, p-value < 0.01) [14]. Similarly, Guo et al. found that patients with NTIS, defined as FT3 < 3.28 pmol/L, had higher 28-day mortality (19.5% vs. 6.4%, p-value = 0.012) [5]. Gutch et al. showed that FT3 was the strongest predictor of ICU mortality, even more than FT4 or APACHE II score [7].

However, while most studies considered FT3 as the most reliable prognostic marker, in this study, FT3 was found to have the lowest AUC, which is against the results of several other studies. For example, Gutch et al. reported an AUC of 0.99 for FT3, higher than that for the APACHE II score [7], while Wang et al. and Chavanda et al. also showed FT3 to be the best predictor for ICU mortality [3,6]. The difference in results may be attributed to variation in study populations, sample size, illness severity, or methods used for hormone measurement.

Regarding T4, in this study’s findings, the association with mortality is in line with some studies, as in the meta-analysis by Vidart et al. that observed a simultaneous reduction in FT3 and FT4, which was linked with higher mortality [14]. Moreover, the prognostic prediction of T4 remains inconsistent, as other studies, such as Chavanda et al., showed no significant difference in FT4 levels between survivors and non-survivors [6]. For TSH, our study did not show any significant association with mortality, which is also consistent with the majority of studies, including the meta-analysis by Vidart et al., where no meaningful difference was found in TSH levels between survivors and non-survivors [14].

The findings of our study showed that NTIS, reflected by low FT3 levels, is strongly associated with clinical indicators of severe disease and adverse outcomes. Patients classified within the low FT3 group had an increased incidence of major complications, including AKI (22% vs. 10.8%), shock (14% vs. 2%), and the need for mechanical ventilation (40% vs. 21.5%, p = 0.007). These results are in line with several previous studies that have shown a strong association between low total T3/FT3 and disease, as in Guo et al.'s study that reported significantly higher APACHE II and SOFA scores among patients with euthyroid sick syndrome (p-value < 0.001 for both) [5]. Chavanda and Mane identified a negative correlation between FT3 and APACHE II (r = −0.4083; p-value = 0.0032) [6]. Similarly, Praveen et al. [15] found higher APACHE II scores in the NTIS group compared with euthyroid patients, and another study in Turkey had observed a strong correlation between FT3 and APACHE II (r = 0.69, p-value = 0.001) [17]. Similar to our results, the study by Bello et al. observed that low FT3 was a significant predictor of prolonged mechanical ventilation [18], while Guo et al. found that serum creatinine is a mortality-related factor, supporting our observed link between NTIS and renal dysfunction [5].

In our cohort, patients with NTIS had elevated CRP and reduced hemoglobin levels. These trends are observed in prior studies demonstrating a consistent inverse relationship between FT3 and systemic inflammation. For instance, Wang et al. found significant negative correlations between FT3 and both CRP (r = −0.408, p-value < 0.001) [3], while Gao et al. found similar negative correlations with CRP (r = −0.66, p-value < 0.001) and IL-6 (r = −0.60, p-value < 0.001) in COVID-19 patients [5].

This study has several limitations that should be considered. First, it was a single-center study with a relatively small sample size, which may limit the generalizability of the findings. Second, due to its observational and non-interventional design, a causal relationship between thyroid dysfunction and mortality cannot be established. The hormonal changes observed may reflect illness severity rather than act as independent predictors. Third, the absence of validated illness severity scoring systems such as APACHE II and SOFA limits adjustment for baseline disease severity; therefore, low FT3 may partly reflect the severity of critical illness rather than serving as an independent prognostic factor. Fourth, thyroid hormones were measured only once upon ICU admission; therefore, dynamic changes during recovery or deterioration were not assessed. Additionally, reverse T3 and cortisol were not evaluated, which could have provided further insight into the neuroendocrine adaptation to stress. Finally, none of the included patients received amiodarone during ICU admission. However, other medications known to influence thyroid function, such as corticosteroids and vasopressor agents, were not systematically recorded, which may represent a residual confounding factor. Despite these limitations, this study provides important local evidence on the prognostic impact of non-thyroidal illness among critically ill patients and highlights the need for larger multicenter studies with longitudinal hormonal assessment and integrated severity scoring.

## Conclusions

In conclusion, the results of this study confirmed that NTIS is common in critically ill patients and that low FT3 is associated with increased mortality and adverse outcomes. Although the predictive accuracy of FT3 for mortality in this cohort appeared less predictable than what was reported elsewhere, the consistent association across multiple studies highlights the importance of recognizing NTIS as a poor prognostic marker. Overall, NTIS is common in the ICU and serves as a marker of illness severity and poor prognosis. Low FT3 was associated with adverse outcomes and may reflect the severity of critical illness rather than acting as a causal determinant. Further studies incorporating validated severity scores are required before establishing FT3 as an independent prognostic marker.
